# AKT and ERK1/2 activation via remote ischemic preconditioning prevents *Kcne2*‐dependent sudden cardiac death

**DOI:** 10.14814/phy2.13957

**Published:** 2019-02-08

**Authors:** Zhaoyang Hu, Jin Liu, Leng Zhou, Xin Tian, Geoffrey W. Abbott

**Affiliations:** ^1^ Laboratory of Anesthesiology & Critical Care Medicine Translational Neuroscience Center West China Hospital Sichuan University Chengdu Sichuan China; ^2^ Department of Anesthesiology West China Hospital Sichuan University Chengdu Sichuan China; ^3^ Bioelectricity Laboratory Department of Physiology and Biophysics School of Medicine University of California Irvine California

**Keywords:** Ischemia reperfusion injury, MinK‐related peptide 1, MiRP1, remote ischemic preconditioning, voltage‐gated potassium channel

## Abstract

Sudden cardiac death (SCD) is the leading global cause of mortality. SCD often arises from cardiac ischemia reperfusion (IR) injury, pathologic sequence variants within ion channel genes, or a combination of the two. Alternative approaches are needed to prevent or ameliorate ventricular arrhythmias linked to SCD. Here, we investigated the efficacy of remote ischemic preconditioning (RIPC) of the limb versus the liver in reducing ventricular arrhythmias in a mouse model of SCD. Mice lacking the *Kcne2* gene, which encodes a potassium channel *β* subunit associated with acquired Long QT syndrome were exposed to IR injury via coronary ligation. This resulted in ventricular arrhythmias in all mice (15/15) and SCD in 5/15 mice during reperfusion. Strikingly, prior RIPC (limb or liver) greatly reduced the incidence and severity of all ventricular arrhythmias and completely prevented SCD. Biochemical and pharmacological analysis demonstrated that RIPC cardioprotection required ERK1/2 and/or AKT phosphorylation. A lack of alteration in GSK‐3*β* phosphorylation suggested against conventional reperfusion injury salvage kinase (RISK) signaling pathway protection. If replicated in human studies, limb RIPC could represent a noninvasive, nonpharmacological approach to limit dangerous ventricular arrhythmias associated with ischemia and/or channelopathy‐linked SCD.

## Introduction

Sudden cardiac death (SCD), the major cause of natural death leads to the loss of an estimated 325,000 adult lives in the U.S. each year. The majority can be attributed to ventricular arrhythmias including ventricular tachycardia or ventricular fibrillation. Most sudden cardiac deaths in young people are caused by an inherited cardiac arrhythmia syndrome, often traceable to a single gene defect, in the absence of detectable structural or functional cardiac abnormality prior to SCD (Fishman et al. 2010; George 2013).

*KCNE2*, one of ~25 genes recognized to associate with risk of cardiac arrhythmia, encodes a single transmembrane domain potassium channel ancillary (*β*) subunit named KCNE2, or MinK‐related peptide 1 (MiRP1) (Abbott et al. 1999). A majority of cardiac arrhythmia cases associated with human KCNE2 sequence variants involve QT prolongation and probably require superimposition of environmental factors such as QT‐prolonging drugs (Abbott et al. 1999; Sesti et al. 2000; Gordon et al. 2008; Abbott 2015). KCNE2 forms heteromeric ion channel complexes with a wide variety of voltage‐gated potassium (Kv) channel pore‐forming *α* subunits in vitro and in vivo (Abbott et al. 1999; Tinel et al. 2000a,2000b; Lewis et al. 2004; Roepke et al. 2006, 2008, 2011; McCrossan et al. 2009; Kanda et al. 2011a,2011b; Abbott 2015), and also with *α* subunits of HCN (pacemaker) channels (Radicke et al. 2008; Nawathe et al. 2013) and L‐type Ca^2+^ channels (Liu et al. 2014).

In addition to Long QT syndrome, sequence variation within or adjoining human *KCNE2* is also associated with early‐onset myocardial infarction (Kathiresan et al. 2009), prevalence of and mortality linked to MI (Szpakowicz et al. 2015), and predisposition to coronary artery disease (Sabater‐Lleal et al. 2014). Reflecting this, in mice, *Kcne2* deletion generates both electrical and systemic substrates that contribute to lethal cardiac rhythm disturbances (Abbott 2012; Hu et al. 2014). The substrates include aging‐associated QTc prolongation, diabetes, anemia, hypercholesterolemia, hyperkalemia, and elevated serum angiotensin II (Hu et al. 2014; Lee et al. 2017). Further, *Kcne2* deletion predisposes mice to atherosclerosis (Lee et al. 2015) and fatty liver (Lee et al. 2016). *Kcne2* deletion also produces a trigger for SCD – when mice were fasted, they became acutely hypoglycemic and hyperkalemic predisposing to AV block and SCD (Hu et al. 2014).

Given the complexity of SCD in the *Kcne2* ^‐/‐^ mouse model and potential parallels to human SCD (*KCNE2*‐associated and otherwise), we sought to investigate potential treatment approaches focused on reducing the incidence of ischemia‐initiated ventricular arrhythmias. Remote ischemic preconditioning (RIPC) is a promising strategy that confers strong cardioprotective effects by applying several short cycles of ischemia–reperfusion stimulus in limbs or visceral organs such as liver, mesentery, intestine or kidney etc. in various animal models (Przyklenk and Whittaker 2011). Here, we examine two different forms of remote ischemic preconditioning (limb and liver), and find that both were effective at preventing lethal ventricular arrhythmias in *Kcne2* ^‐/‐^ mice. By biochemically and pharmacologically dissecting the mechanisms involved, we identify the signaling proteins required for successful prevention by RIPC of *Kcne2*‐dependent ischemia‐provoked arrhythmias.

## Materials and Methods

### Animals

Adult female *Kcne2* ^+/+^ and *Kcne2* ^‐/‐^ C57BL/6 mice of 13 months of age were generated as previously described (Hu et al. 2014) and the study was approved by the Institutional Animal Care and Use Committee of Sichuan University (Sichuan, China) (Permit Number: 2015035A). The study was carried out in accordance with the recommendations in the Guide for the Care and Use of Laboratory Animals of the National Institutes of Health (NIH Publication 8th edition, 2011). All mice were housed under a 12‐h light–dark cycle at 20–25°C and humidity of 60 ± 5%.

### Experimental protocol and surgical procedures

**Figure 1:**
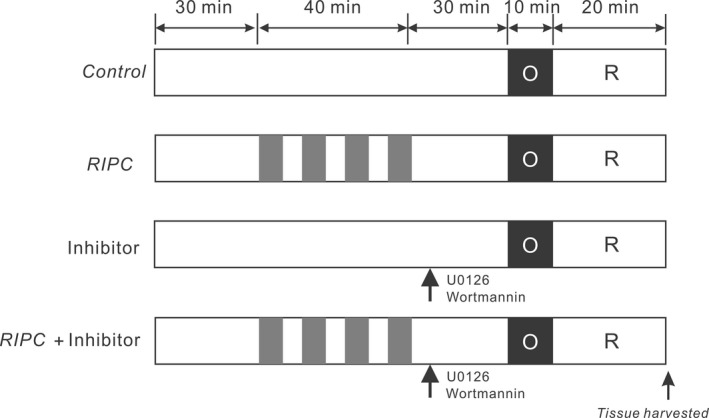
Experimental protocols. Remote ischemic preconditioning (RIPC) of liver or limb was induced by four cycles of 5 min of ischemia with 5 min intermittent reperfusions. Pharmacological inhibitors were administered as a bolus 30 min prior to myocardial ischemia. Mice of either genotype were subjected to 10‐min left main coronary artery occlusion followed by 20‐min reperfusion.

Experimental protocols are summarized in Figure 1. All mice were anesthetized with intraperitoneal injection of sodium pentobarbital (50 mg/kg) for anesthesia. The loss of the corneal reflex and the lack of response to toe‐pinching were indicators for monitoring anesthesia adequacy. Mice were artificially ventilated with a mouse ventilator (Harvard Apparatus, Holliston, MA) with a tidal volume of 250 *μ*L at a rate of 150 strokes/min. After thoracotomy, the main left coronary artery was ligated for 10 min with a 9‐0 silk ligature (Ethicon, Somerville, NJ) close to its origin; the suture was then released, followed by 20 min of reperfusion for production of coronary artery ischemia reperfusion injury. For remote liver preconditioning, as we previously reported (Hu et al. 2016, 2017; Yang et al. 2017), laparotomy was performed and the portal vein, hepatic arterial and venous trunk were identified and occluded using a noninvasive vascular clamp. Four cycles of 5 min of liver ischemia with 5 min intermittent reperfusion were conducted for liver ischemic preconditioning (RIPC‐liver). Remote limb ischemic preconditioning (RIPC‐limb) was achieved with four cycles of 5‐min ischemia/5‐min reperfusion in the hind limbs by placing an elastic rubber band tourniquet on the proximal part of the limbs. Liver (or limb) ischemia was confirmed by a change in the liver (or limb) color, which returned to pink after reperfusion. Thirty minutes after ischemic preconditioning stimulus, mice were subjected to myocardial ischemia insults. To identify the requirement of specific proteins involved in RIPC induced‐anti‐arrhythmic effects, AKT inhibitor wortmannin (15 *μ*g/kg) or ERK1/2 inhibitor U0126 (0.6 mg/kg) (Sigma, St. Louis, MO) solubilized in dimethyl sulfoxide (DMSO) were intravenously bolus‐injected into the mouse femoral vein 30 min before left main coronary artery ligation. Mouse body temperature was maintained with a heating blanket. At the end of the experiment, mice were euthanized with an overdose of sodium pentobarbital (200 mg/kg, i.p.) and death was monitored via cardiac activity and respiration. The coronary artery was reoccluded, and 1% Evans blue (Sigma) was injected into the left ventricular cavity to depict the ischemic area at risk (AAR). AAR tissue was stored at −80°C for subsequent western blotting analysis.

### Electrocardiography analysis

ECG parameters were recorded throughout the experiments using a PowerLab/8sp system (AD Instruments, Colorado Springs, CO). A standard limb lead 2 configuration system was used and needle electrodes were attached to the mice underneath the skin. LabChart 7.2.1 software (AD Instruments) was used to analyze the following arrhythmia events recorded during the 20 min of reperfusion injury period: (1) Number of mice with any arrhythmia ‐ AV block (AVB); sudden cardiac death (SCD); ventricular tachycardia (VT); polymorphic VT (PVT); or sustained VT (>10 sec) (SVT). (2) Duration of VT. (3) Start time of the first run of ventricular tachycardia.

### Western blotting

Frozen hearts were homogenized in RIPA buffer (50 mmol/L Tris‐HCl (pH7.4), 150 mmol/L NaCl, 1% NP‐40, 1 mmol/L EDTA, 0.25% sodium deoxycholate, phosphatase inhibitor cocktail (Sigma), and a protease inhibitor cocktail (Sigma), followed by centrifugation at 10,000 ***g*** for 10 min. The supernatant was retained for electrophoresis. Protein concentration was determined using BCA (Pierce, Rockford, IL). 15 *μ*g of protein was loaded in each lane, resolved on a 12% SDS‐PAGE gel and then transferred onto nitrocellulose membranes (VWR, Batavia, IL). The membrane was probed with primary antibodies raised against: phosphorylated extracellular signal‐regulated kinase1/2 (ERK1/2) (Thr202/Tyr204), (p‐ERK), total ERK1/2, phosphorylated Akt(ser473)(p‐AKT), total Akt, phosphorylated glycogen synthase kinase‐3*β*(Ser9), (p‐GSK‐3*β* Ser9), total GSK‐3*β*, phosphorylated STAT‐3(Tyr705)(p‐STAT‐3) and total STAT‐3 (all 1:1000 dilution, from Cell Signaling, Danvers, MA). After overnight incubation, the membranes were incubated with horseradish peroxidase (HRP)‐conjugated goat anti‐rabbit IgG secondary antibody (all 1:5000 dilution, from Bio‐Rad, Hercules, CA) before being visualized by chemiluminescence ECL (Millipore, Billerica, MA). Images were captured using an Amersham Imager 600 system (GE Healthcare, Little Chalfont, UK). Image processing and band density analysis were performed using ImageJ Data Acquisition Software (National Institutes of Health, Bethesda, MD). Phosphorylation‐signal densities were each normalized to the corresponding total protein‐signal densities.

### Statistical analysis

All values were tested for normal distribution using the Kolmogorov–Smirnov test and are expressed as mean ± SEM. Fisher's exact test was employed to compare the number of mice falling into one of the two groups. Differences among groups over two were analyzed using one‐way ANOVA. Homogeneity of variance was tested using Levene's test. The Newman–Keuls test was examined post hoc for multiple comparisons if variances were equal; or, Dunnett's T3 test was used. All *P*‐values were two‐sided. *P *<* *0.05 was used to indicate statistical significance.

## Results

### The effect of remote conditioning on postischemic ventricular arrhythmias in Kcne2^‐/‐^ mice

**Figure 2:**
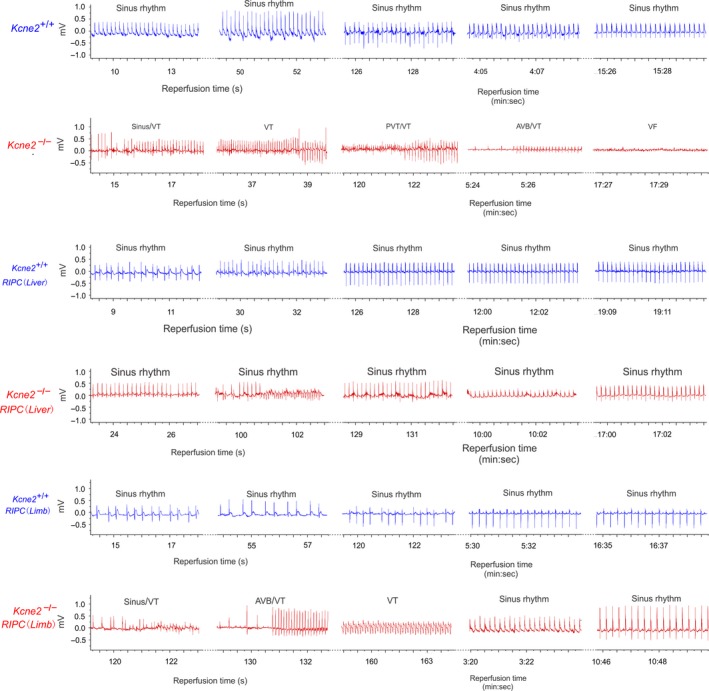
Remote ischemic preconditioning (RIPC) protects against *Kcne2*‐dependent ventricular arrhythmias. Exemplar surface ECGs of *Kcne2* ^*+/+*^ and *Kcne2* ^*‐/‐*^ mice in the presence or absence of liver or limb preconditioning (RIPC) during the 20 min of cardiac reperfusion period (*n *=* *8–15). AVB, AV block, VT, ventricular tachycardia, PVT indicates polymorphic VT.

**Figure 3:**
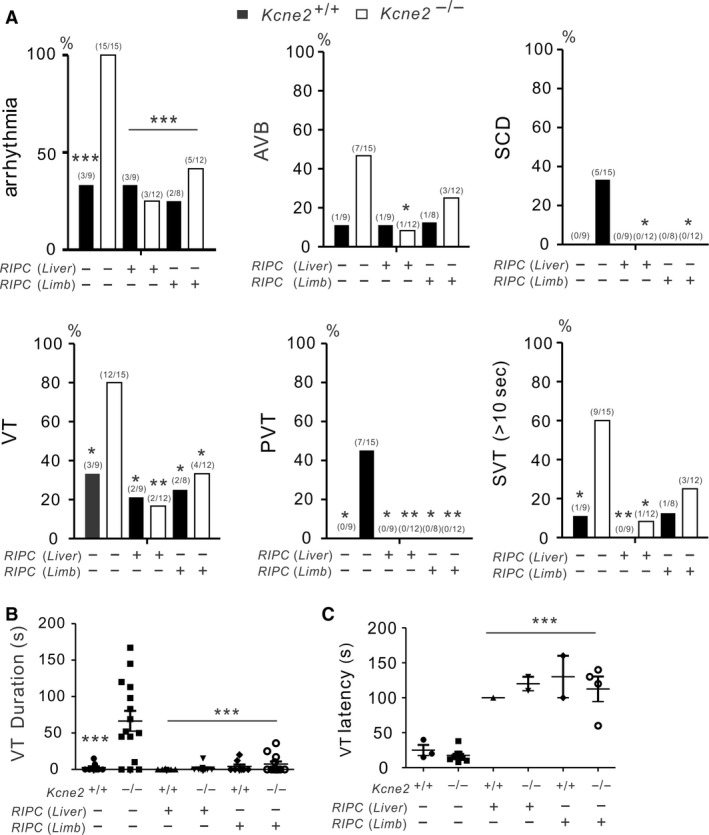
Remote ischemic preconditioning (RIPC) prevents lethal Kcne2‐dependent ventricular arrhythmias. (A, B) Quantification of cardiac arrhythmia incidence in *Kcne2* ^*+/+*^ and *Kcne2* ^*‐/‐*^ mice with or without RIPC (Liver or Limb) treatment (*n *=* *9–15). Numbers of mice per category are indicated in parentheses. (A) Arrhythmia, all arrhythmia classes pooled; AVB, AV block; SCD, sudden cardiac death; VT, ventricular tachycardia; PVT, polymorphic VT; SVT, sustained ventricular tachycardia (over 10 sec); *P < 0.05, **P < 0.01, ***P < 0.001 versus *Kcne2* ^*‐/‐*^ mice without RIPC treatment. (B) Mean VT durations for *Kcne2* ^*+/+*^ and *Kcne2* ^*‐/‐*^ mice with or without RIPC (Liver or Limb) treatment (*n *=* *8–15). Mice without VT were indicated as 0 sec duration. ***P < 0.001, versus *Kcne2* ^*‐/‐*^ mice without RIPC treatment (by one‐way ANOVA). (C) Latency to first run of VT after the onset of reperfusion in *Kcne2* ^*+/+*^ and *Kcne2* ^*‐/‐*^ mice with or without RIPC (Liver or Limb) treatment (*n *=* *9–15). ***P < 0.001, versus *Kcne2* ^*‐/‐*^ mice without RIPC treatment (by one‐way ANOVA).

To explore the role of *Kcne2* deletion on RIPC‐induced antiventricular arrhythmias, all *Kcne2* ^+/+^ and *Kcne2* ^‐/‐^ mice with or without RIPC stimulus were exposed to a 10‐min left main coronary ligation followed by 20‐min reperfusion. Representative ECG tracings from *Kcne2* ^+/+^ and *Kcne2* ^‐/‐^ mice postIR are presented in Figure 2. Consistent with our previous findings (Hu et al. 2014), *Kcne2* deletion increased the predisposition to ventricular arrhythmogenesis during the postischemic reperfusion period. Strikingly, RIPC stimulus (liver or limb) exerted strong antiarrhythmic action as illustrated in Figure 2, with quantification shown in Figure 3 and described below.

Thus, all *Kcne2* ^‐/‐^ mice (15 out of 15, *P* = 0.0006 vs. *Kcne2* ^*+/+*^ mice) developed arrhythmias throughout reperfusion including ventricular tachycardia (VT), atrioventricular block (AVB), polymorphic ventricular tachycardia (PVT), or sustained ventricular tachycardia (SVT) exceeding 10 sec duration. However, RIPC‐treated *Kcne2* ^‐/‐^ mice were less susceptible to lethal ventricular arrhythmia. Only three out of 12 RIPC‐liver‐treated and five out of 12 RIPC‐limb‐treated *Kcne2* ^‐/‐^ mice exhibited arrhythmia (*P* < 0.001 vs. *Kcne2* ^‐/‐^ control mice), while the remainder remained in sinus rhythm (Fig. 3A).

We found that 47% (7/15) of the *Kcne2* ^‐/‐^ mice developed AVB, with an increased incidence (5/15 mice) of SCD during reperfusion. Although limb ischemic conditioning (3/12, 25%) was not as effective as liver preconditioning (1/12, 8%; *P* = 0.0433 vs. *Kcne2* ^‐/‐^ control mice) in reducing the incidence of AVB, both liver and limb conditioning exerted a striking cardioprotective effect against SCD in *Kcne2* ^‐/‐^ mice (5 death/15 *Kcne2* ^‐/‐^ control mice, compared to 0 death/12 RIPC‐liver‐ or limb‐treated *Kcne2* ^‐/‐^ mice; *P* = 0.047) (Fig. 3A).

In addition, VT was more prevalent in *Kcne2* ^‐/‐^ mice (12/15) than in RIPC‐liver treated *Kcne2* ^‐/‐^ mice (2/12, *P* = 0.0018) or RIPC‐limb treated *Kcne2* ^‐/‐^ mice (4/12, *P* = 0.022). Notably, none of the RIPC‐treated *Kcne2* ^‐/‐^ mice exhibited PVT (Liver, 0/12, *P* = 0.0081; Limb, 0/12, *P* = 0.0081 vs. *Kcne2* ^‐/‐^ control mice). In contrast, seven out of 15 untreated *Kcne2* ^‐/‐^ mice showed solely polymorphic VT (*P* = 0.0223 vs. *Kcne2* ^*+/+*^ mice). Meanwhile, liver ischemic preconditioning resulted in a low incidence of SVT (>10 sec) (1/12) when compared to *Kcne2* ^‐/‐^ mice without any preconditioning stimulus (9/15, *P* = 0.014). There was a similar pattern for SVT induction in RIPC‐limb‐treated *Kcne2* ^‐/‐^ mice, however, no statistical significance was detected (*P* > 0.05) (Fig. 3A).

*Kcne2* deletion prolonged the mean VT duration from 2.6 ± 1.7 sec to 66.5 ± 13.8 sec compared to their wild‐type littermates (*P* < 0.001). However, *Kcne2* ^‐/‐^ mice with liver stimulus (1.4 ± 1.2 sec) or limb stimulus (7.3 ± 3.5 sec) had markedly shorter mean VT duration than that of non RIPC‐treated *Kcne2* ^‐/‐^ mice (*P* < 0.0001) (Fig. 3B). Furthermore, RIPC delayed the onset of the first run of VT after commencing reperfusion in both genotypes compared to *Kcne2* ^*‐/‐*^ mice without RIPC treatment (*P* < 0.001) (Fig. 3C).

### The effect of RIPC on RISK pathway protein phosphorylation in Kcne2^‐/‐^ mice

**Figure 4:**
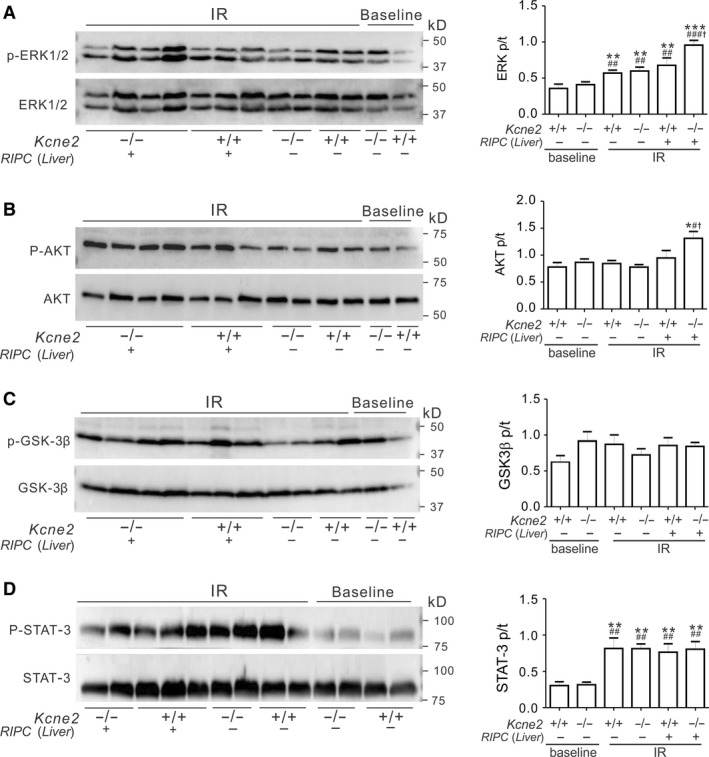
Liver remote ischemic preconditioning (RIPC) stimulates ventricular ERK1/2 and AKT phosphorylation in Kcne2^‐/‐^ mice post cardiac IR injury. (A‐D) *Left,* representative western blots of phospho‐(p) ERK1/2 and total (t)ERK1/2 (A), phospho‐(p) AKT and total (t) AKT (B), phospho‐(p) GSK3*β* and total (t) GSK3*β* (C), phospho‐(p) STAT‐3 and total (t) STAT‐3 (D) from *Kcne2* ^*+/+*^ and *Kcne2* ^*‐/‐*^ mice with or without RIPC(Liver) treatment; one mouse per lane. *Right,* mean ratio of band densities of pERK/tERK (A, **P < 0.01, ***P < 0.001, vs. baseline *Kcne2* ^*+/+*^ mice, ^##^ *P < *0.01, ^###^ *P < *0.001 vs. baseline *Kcne2* ^*‐/‐*^ mice, ^†^ *P < *0.05 vs. *Kcne2* ^*+/+*^ mice after RIPC(Liver) treatment), pAKT/tAKT (B, *P < 0.05, vs. baseline *Kcne2* ^*+/+*^ mice, ^#^ *P < *0.05 vs. baseline *Kcne2* ^*‐/‐*^ mice, ^†^ *P < *0.05 vs. *Kcne2* ^*+/+*^ mice after RIPC(Liver) treatment), pGSK3*β*/tGSK3*β* (C), pSTAT‐3/tSTAT‐3 (D, **P < 0.01, vs. baseline *Kcne2* ^*+/+*^ mice, ^##^ *P < *0.01, vs. baseline *Kcne2* ^*‐/‐*^ mice). *n *=* *4–5 each group.

To explore the mechanisms underlying the RIPC‐induced cardioprotective effect, and the possible differences between liver and limb preconditioning, we investigated whether or not *Kcne2* deletion and/or RIPC altered phosphorylation levels (as a means to quantify specific signaling pathway activation) of proteins in the reperfusion injury salvage kinase (RISK) pathway, specifically ERK1/2, AKT, and GSK‐3*β*; and the survivor activating factor enhancement (SAFE) pathway, specifically STAT‐3. The total ERK1/2, AKT, and GSK‐3*β* levels in RISK pathway, as well as the total STAT‐3 levels were not different in all tested groups. We normalized the phosphorylation level of each protein to its corresponding total protein level (Fig. 4).

Effects of liver RIPC are described first. Compared with baseline ventricles, I/R injury elevated the phosphorylation levels of ERK1/2 (*P* < 0.01). Liver RIPC further increased ERK1/2 phosphorylation; increasing the ratio of phosphorylated (p) to total (t) ERK1/2 in *Kcne2* ^‐/‐^ mice to more than double that of baseline *Kcne2* ^‐/‐^ mice (*P* < 0.001) (Fig. 4A). We also observed ~50% increased ERK1/2 phosphorylation (Fig. 4A) and 39% increased AKT phosphorylation (Fig. 4B) in *Kcne2* ^‐/‐^ mouse ventricles after liver preconditioning compared with RIPC‐treated *Kcne2* ^+/+^ mice (*P* < 0.05). Meanwhile, we saw no difference in the ratio of ventricular phosphorylated to total GSK‐3*β* (Ser9), between genotypes either before or after RIPC treatment post I/R, or in RIPC‐liver‐treated versus untreated mice (Fig. 4C, *P* > 0.05). The ratio of phosphorylated to total STAT‐3 more than doubled in mice of either genotype postIR when compared to their baseline values (Fig. 4D, *P* < 0.01), however, there were no RIPC‐liver‐ or genotype‐dependent differences in STAT‐3 phosphorylation levels (Fig. 4D, *P* > 0.05).

**Figure 5:**
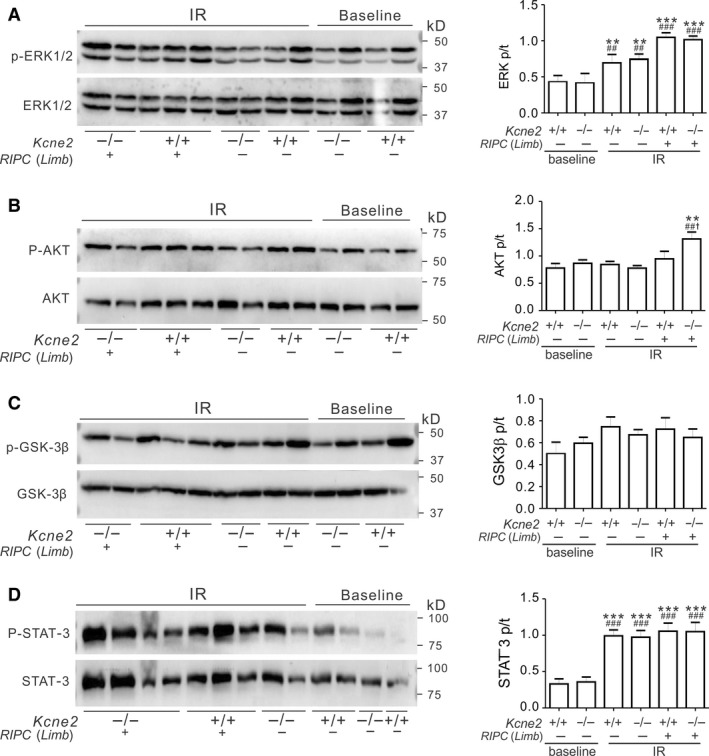
Limb remote ischemic preconditioning (RIPC) stimulates ventricular AKT phosphorylation in Kcne2^‐/‐^ mice post cardiac IR injury. (A–D) *Left,* typical blots of phospho‐(p) ERK1/2 and total (t)ERK1/2 (A), phospho‐(p) AKT and total (t) AKT (B), phospho‐(p) GSK3*β* and total (t) GSK3*β* (C), phospho‐(p) STAT‐3 and total (t) STAT‐3 (D) from *Kcne2* ^*+/+*^ and *Kcne2* ^*‐/‐*^ mice with or without RIPC(Limb) treatment; one mouse per lane. *Right,* mean ratio of band densities of pERK/tERK (A, *P < 0.05, ***P < 0.001, vs. baseline *Kcne2* ^*+/+*^ mice, ^#^ *P < *0.05, ^###^ *P < *0.001 vs. baseline *Kcne2* ^*‐/‐*^ mice, ^†^ *P < *0.05 vs. *Kcne2* ^*+/+*^ mice after RIPC(Limb) treatment), pAKT/tAKT (B, **P < 0.01, vs. baseline *Kcne2* ^*+/+*^ mice, ^##^ *P < *0.01, vs. baseline *Kcne2* ^*‐/‐*^ mice, ^†^ *P < *0.05 vs. *Kcne2* ^*+/+*^ mice after RIPC(Limb) treatment), pGSK3*β*/tGSK3*β* (C), pSTAT‐3/tSTAT‐3 (D, ***P < 0.01 vs. baseline *Kcne2* ^*+/+*^ mice*,* ^###^ *P < *0.01 vs. baseline *Kcne2* ^*‐/‐*^ mice). *n *=* *4–5 each group.

Phosphorylation of the above‐mentioned four proteins in the RISK and SAFE pathways was also quantified in mice treated with limb ischemic preconditioning. Prior RIPC‐limb significantly increased phosphorylation of ERK1/2 in both genotypes, and of AKT only in *Kcne2* ^‐/‐^ mice (the latter also showing higher AKT phosphorylation versus RIPC‐limb treated Kcne2^+/+^ mice) following cardiac reperfusion (*P* < 0.05) (Fig. 5A,B). No genotype‐ or RIPC‐limb‐dependent differences were detected regarding the phosphorylation levels of GSK‐3*β* or STAT‐3, although phosphorylation of the latter was again more than doubled by I/R injury (Fig. 5C,D).

### Pharmacological inhibition of RISK pathway impairs the antiarrhythmic action of remote conditioning

**Figure 6:**
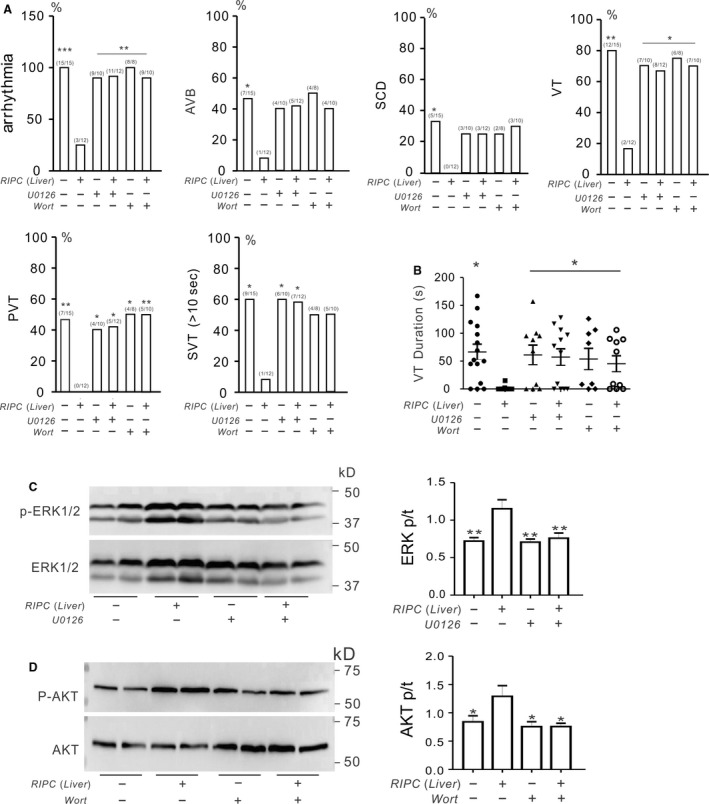
AKT and ERK1/2 activation is essential for prevention of SCD by remote ischemic preconditioning (RIPC)‐liver in Kcne2^‐/‐^ mice postIR. (A) The change of cardiac arrhythmia incidence in the absence (−) or presence (+) of liver I/R preconditioning stimulus when applying ERK inhibitor U0126 or AKT inhibitor Wortmannin (Wort) in *Kcne2* ^*‐/‐*^ mice (*n *=* *8–15 per group). Numbers of mice per category are indicated in parentheses. Values for *Kcne2* mice without inhibitors are repeated from Fig. 2 for comparison. AVB, AV block; SCD, sudden cardiac death; VT, ventricular tachycardia; PVT, polymorphic VT; SVT, sustained ventricular tachycardia over 10 sec. *P < 0.05,**P < 0.01, versus RIPC‐treated *Kcne2* ^*‐/‐*^ mice post I/R. (B) Durations of VT for RIPC (Liver)‐treated or nontreated *Kcne2* ^*‐/‐*^ mice in the presence (+) or absence (−) of ERK inhibitor U0126 or AKT inhibitor Wortmannin (Wort) application. Mice without VT were indicated as 0 sec duration. Values for *Kcne2* ^*‐/‐*^ mice without inhibitors are repeated from Figre 2 for comparison. *P < 0.05, versus RIPC‐treated *Kcne2* ^*‐/‐*^ mice post I/R. (C) *Left,* typical blot of phospho‐(p) ERK1/2 and total (t)ERK1/2 from *Kcne2* ^*‐/‐*^ mice with or without RIPC(Liver) treatment in the presence(+) or absence(−) of ERK inhibitor U0126 or AKT inhibitor Wortmannin (Wort) application. *Right,* mean ratio of band densities of pERK/tERK. **P < 0.01, versus *Kcne2* ^*‐/‐*^ mice after RIPC (Liver) treatment without inhibitors, *n* = 4–5 each group. (D) *Left,* typical blot of phospho‐(p) AKT and total (t)AKT from *Kcne2* ^*‐/‐*^ mice with or without RIPC(Liver) treatment in the presence(+) or absence(−) of ERK inhibitor U0126 or AKT inhibitor Wortmannin (Wort) application. *Right,* mean ratio of band densities of pAKT/tAKT(*n* = 4–5 each group).*P < 0.01, versus *Kcne2* ^*‐/‐*^ mice after RIPC (Liver) treatment without inhibitors.

ERK1/2 and/or AKT were phosphorylated after liver preconditioning treatment preferentially in *Kcne2* null mice. To further explore the respective roles of these signaling molecules in RIPC‐liver‐induced cardioprotection, we first applied pharmacological inhibitors (U0126 to inhibit ERK1/2; wortmannin to inhibit AKT) after liver preconditioning but prior to cardiac I/R. U0126 and wortmannin each disrupted RIPC‐liver‐induced cardioprotection in *Kcne*2^‐/‐^ mice, such that postI/R arrhythmia incidence and severity were significantly increased in all inhibitor‐treated *Kcne*2^‐/‐^ mice, and VT durations prolonged, to levels similar to those of *Kcne2* ^‐/‐^ mice without RIPC stimulus (*P* < 0.05, or *P* < 0.01 vs. RIPC‐treated *Kcne*2^‐/‐^ mice, Fig. 6A,B). ERK and AKT were phosphorylated in RIPC‐treated *Kcne*2^‐/‐^ mice; however, this RIPC‐induced phosphorylation was blocked by U0126 (*P < *0.01). Furthermore, wortmannin diminished AKT phosphorylation compared to *Kcne*2^‐/‐^ mice exposed to RIPC alone, to a level similar to nontreated *Kcne*2^‐/‐^ hearts post I/R (*P < *0.05, Fig. 6C,D).

**Figure 7:**
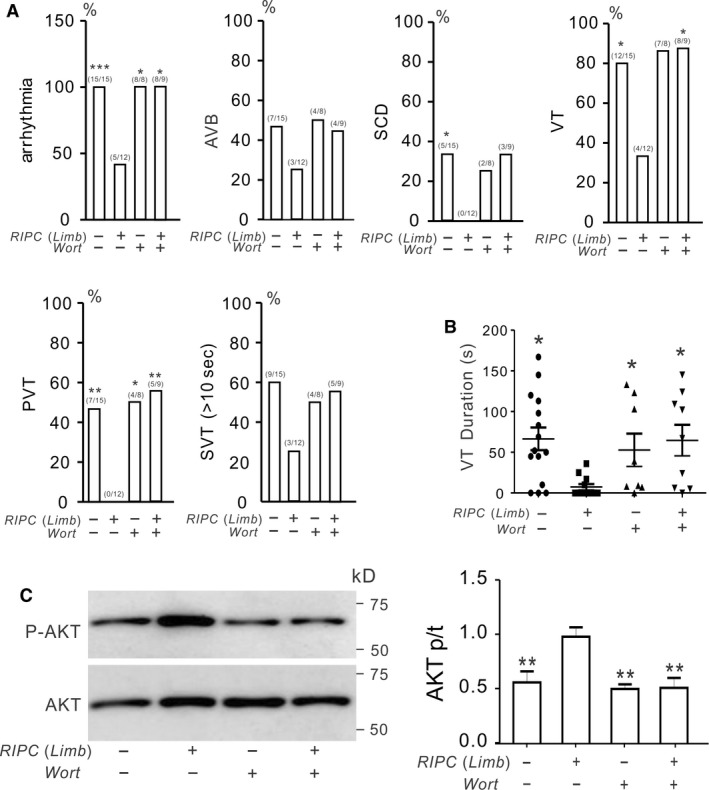
AKT phosphorylation is essential for remote ischemic preconditioning (RIPC)‐limb‐induced antiarrhythmic activity in Kcne2^‐/‐^ mice post‐IR. (A) The effect of inhibiting AKT (Wortmannin, Wort) on postreperfusion arrhythmia characteristics in *Kcne2* ^*‐/‐*^ mice in the absence (−) or presence (+) of limb I/R preconditioning stimulus. Values in parentheses indicate the numbers of mice per category. *n *=* *8–15 each group. Values for *Kcne2* mice without inhibitor are repeated from Figure 2 for comparison. AVB, AV block; SCD, sudden cardiac death; VT, ventricular tachycardia; PVT, polymorphic VT; SVT, sustained ventricular tachycardia over 10 sec. (B) Durations of VT for RIPC‐Limb‐treated or nontreated *Kcne2* ^*‐/‐*^ mice in the presence (+) or absence (−) of AKT inhibitor Wortmannin (Wort) application. Mice without VT were indicated as 0 sec duration. Values for *Kcne2* ^*‐/‐*^ mice without inhibitors are repeated from Figure 2 for comparison. *P < 0.05 versus *Kcne2* ^*‐/‐*^ mice after RIPC‐limb treatment without inhibitor. (C) *Left,* typical blot of phospho‐(p) AKT and total (t) AKT from *Kcne2* ^*‐/‐*^ mice with or without RIPC‐limb treatment in the presence (+) or absence (−) of AKT inhibitor Wortmannin (Wort) application. *Right,* mean ratio of band densities of pAKT/tAKT (*n *=* *4–5 each group).*P < 0.01, versus *Kcne2* ^*‐/‐*^ mice after RIPC‐limb treatment without inhibitors.

We next tested the effect of inhibiting AKT in RIPC‐limb‐induced cardioprotection in *Kcne2* ^‐/‐^ mice. As expected, RIPC‐limb‐treated *Kcne*2^‐/‐^ mice were less susceptible to postI/R arrhythmia compared to nontreated *Kcne*2^‐/‐^ mice, as seen in Figures 3 and 7A. However, pretreatment with wortmannin eliminated the antiarrhythmia effect afforded by limb preconditioning. Thus, we observed a similar incidence in *Kcne2* ^‐/‐^ mice receiving no treatment post I/R, and *Kcne2* ^‐/‐^ mice given both wortmannin and RIPC‐limb treatment, of arrhythmogenesis, AVB, SCD, VT, PVT, and SVT (all *P* > 0.05; Fig. 7A). AKT inhibition also increased VT duration compared to *Kcne2* ^‐/‐^ mice exposed to RIPC‐limb preconditioning alone (*P *<* *0.05; Fig. 7B). Correspondingly, wortmannin prevented the RIPC‐limb‐induced increase in AKT phosphorylation (Fig. 7C).

## Discussion

Sudden cardiac death claims 1000 lives per day in the United States alone, and particularly in the case of monogenic arrhythmia syndromes such as LQTS can affect all ages. New therapeutic approaches are needed to improve survival rates after cardiac arrest and reduce the incidence of cardiac arrest in the context of I/R injury.

Remote ischemic preconditioning is a promising strategy that confers strong cardioprotective effects via application of several short cycles of ischemia–reperfusion stimulus in limbs or visceral organs such as the liver, mesentery, intestine or kidney, in various animal models (Przyklenk and Whittaker 2011). We and others have shown that RIPC‐limb (Hausenloy and Yellon 2009) and RIPC‐liver (Yang et al. 2017) can be cardioprotective by reducing infarct damage following myocardial ischemia/reperfusion injury. RIPC may stimulate and upregulate endogenous protective mechanisms, for instance signaling molecules that can travel from the remote tissue to the target organ (heart) and render it resistant to subsequent ischemia‐reperfusion injury by activating, in this case, AKT and ERK1/2. Although the mechanisms underlying RIPC remain unclear, one suggestion is that RIPC causes hypoxic inhibition of PHD2, an oxygen‐sensing enzyme, which then in turn therapeutically increases levels of circulating kynurenic acid (Olenchock et al. 2016).

The mechanisms involved in this protective phenomenon might include multiple effectors, such as sympathetic/parasympathetic mechanisms, as bilateral vagotomy was previously shown to abolish cardioprotection induced by RIPC (Basalay et al. 2018). In addition, increased AKT activity stimulates Kv1.5 potassium channel protein expression at the cell surface (Warsi et al. 2015). This would be expected to be antiarrhythmic in models such as the *Kcne2* ^‐/‐^ mouse, in which Kv1.5 protein at the intercalated discs is reduced because KCNE2 is required for its efficient trafficking there (Roepke et al. 2008). Furthermore, ERK1/2 is implicated in the activation of potassium channels (Teos et al. 2008), L‐type calcium channels (Cheng et al. 2016) and the Na^+^/H^+^ exchanger (Fliegel 2001), all of which are potential targets for antiarrhythmic actions.

In line with several reports showing that limb ischemic preconditioning could raise the tolerance to myocardial reperfusion‐induced arrhythmia (Dow et al. 2012), in a rat model of I/R‐induced ventricular arrhythmia, we also previously found that liver ischemic preconditioning protected hearts by reducing the incidence of ventricular arrhythmia and SCD (Hu et al. 2016, 2017). It is important to note that cardiac cell death occurs after 30–40 min of severe ischemia, thus, our 10 min ligation does not in itself cause significant cardiomyocyte death. Therefore, the observed effects are not secondary to an effect of RIPC increasing cell survival.

Despite its convincing roles in cardioprotection, the influence of RIPC on lethal ventricular arrhythmogenesis linked to disruption of ion channel genes has remained largely unexplored. In addition, little attention has been paid to signaling pathways that might be involved in postI/R arrhythmogenesis.

The reperfusion injury salvage kinase (RISK) signaling pathway including protein kinase B (AKT) and extracellular signal‐regulated kinases (ERK1/2) signaling molecules and the SAFE pathway including signal transducer and activator of transcription 3 (STAT‐3) are intrinsic prosurvival signaling cascades, in which proteins are phosphorylated and thus activated upon remote ischemic preconditioning, limiting infarct size (Tamareille et al. 2011; Yang et al. 2017). Previously, we found that RISK pathway activation stemming from liver RIPC reduced the severity of I/R‐induced ventricular arrhythmias in a rat model. We also observed increased phosphorylation of myocardial kinases ERK1/2 and further GSK‐3*β* at Ser9 but not Tyr216 in the rat model (Hu et al. 2016). However, this prior work did not establish whether the more practical RIPC‐limb was effective, nor did it address the efficacy of RIPC in protecting against increased arrhythmia predisposition linked to a gene defect.

In the present study, we find a clear ability of RIPC (either limb or liver) to completely prevent SCD, and limit the incidence and severity of ventricular arrhythmias and AV block, in a mouse model of acquired LQTS, the *Kcne2* ^‐/‐^ mouse line. Activation of both AKT and ERK1/2 was essential for RIPC‐induced cardioprotection with respect to limiting ventricular arrhythmias and AVB, and preventing SCD. In contrast to our previous work on rats, here we found in mice that this activity did not require differential inactivation of GSK‐3*β*, phosphorylation of which was unaltered by either limb or liver RIPC and was independent of genotype. This suggests that the cardioprotective effect of RIPC‐induced AKT and ERK1/2 phosphorylation in this study involved, at least in part, pathways outside of RISK. Elucidation of the specific pathways in question requires further study. The lack of involvement of GSK‐3*β* is consistent with previous findings for preconditioning or postconditioning of mouse heart (Nishino et al. 2008)**.** Overall, the results of our study suggest that RIPC, particularly the noninvasive limb RIPC, could be useful in some scenarios in preventing dangerous ventricular arrhythmias during ischemia reperfusion. Given that many antiarrhythmic drugs are paradoxically also proarrhythmic (Zipes 1987), nonpharmacological approaches are certainly warranted. Alternatively, pharmacological stimulation of ERK1/2 and/or AKT might also be safer in some contexts than classical antiarrhythmics, whilst also being effective.

There are several caveats in terms of how translatable the results are to human SCD. The mouse heart beats ten times faster, and is much smaller than the human heart. Abnormal waveforms and arrhythmic electrical activity therefore propagate very differently in the two. In addition, mouse and human heart tissue each contain a different array of ion channels, with the primary differences thought to be in the Kv channels. This is balanced somewhat by previous findings that KCNE2 disruption impairs function of the major repolarizing Kv currents in both mouse and human heart (Abbott et al. 1999; Zhang et al. 2001; Roepke et al. 2008; Abbott 2015), but nevertheless is a major caveat of studies involving most animal models, and especially those in mice and rats. Finally, RIPC‐limb (or a pharmacological surrogate) would need to be applied with the appropriate timing for therapeutic efficacy in humans, and might not be as effective as it is in mice. However, recent trials showed that preventive short episodes of arm ischemia reduce platelet activity, and inflammatory and cardiac tissue damage markers, prior to radiofrequency catheter ablation for paroxysmal atrial fibrillation (Han et al. 2016; Kosiuk et al. 2016). This points to one possible useful application of RIPC‐limb, in preventing ventricular arrhythmias during surgical procedures that involve ventricular IR, or perhaps if applied immediately after an unplanned ischemic event but before reperfusion. The impact on efficacy of timing of RIPC with respect to ischemia and reperfusion will be assessed in future studies.

## Conflict of Interest

None declared.
